# Consensus on the definition of locoregional recurrence of colon cancer: protocol for an international Delphi study

**DOI:** 10.1136/bmjopen-2026-118765

**Published:** 2026-06-30

**Authors:** Eva Rademaker, Anne E Petersen, Eva Angenete, Alvaro Arjona-Sánchez, Stefan Benz, Claus A Bertelsen, Johanne G Bloemen, Wim Ceelen, Esther C J Consten, Deena Harji, Ignace H J T de Hingh, Olivier Glehen, Ben Griffiths, Helene Perregaard, Jeanine M L Roodhart, Martin Rutegård, Henk M W Verheul, Johannes H W de Wilt, Henderik L van Westreenen, Des C Winter, Jurriaan B Tuynman, Joep P M Derikx, Pieter J Tanis

**Affiliations:** 1 Department of Surgery, Isala Zwolle, Zwolle, Netherlands; 2 Department of Surgical Oncology and Gastrointestinal Surgery, Erasmus MC Cancer Center, Rotterdam, Netherlands; 3 Department of Paediatric Surgery, Emma Children’s Hospital, University of Amsterdam, Amsterdam University Medical Centres, Amsterdam, Netherlands; 4 Amsterdam Gastroenterology Endocrinology Metabolism Institute, Amsterdam University Medical Centres, Amsterdam, Netherlands; 5 Tytgat Institute for Liver and Intestinal Research, Amsterdam UMC, University of Amsterdam, Amsterdam, Netherlands; 6 Department of Surgery, SSORG-Scandinavian Surgical Outcomes Research Group, Institute of Clinical Sciences, University of Gothenburg Sahlgrenska Academy, Gothenburg, Sweden; 7 Department of Surgery, Region Västra Götaland, Sahlgrenska Universitetssjukhuset, Gothenburg, Sweden; 8 Unit of Surgical Oncology, Reina Sofia University Hospital, Córdoba, Spain; 9 GE09 Research in Peritoneal and Retroperitoneal Oncologic Surgery Group, Maimonides Biomedical Research Institute of Cordoba, Córdoba, Spain; 10 Klinikum Sindelfingen-Boblingen Kliniken Sindelfingen, Sindelfingen, Germany; 11 Department of Surgery, Copenhagen University Hospital, Hillerød, North Zealand, Denmark; 12 Department of Clinical Medicine, University of Copenhagen Faculty of Health and Medical Sciences, Copenhagen, Denmark; 13 Department of Surgery, Catharina Hospital, Eindhoven, Netherlands; 14 Department of GI Surgery, Cancer Research Institute Ghent (CRIG), University Hospital Ghent, Ghent, Belgium; 15 Department of Human Structure and Repair, Ghent University, Ghent, Belgium; 16 Department of Surgery, University of Groningen, University Medical Centre Groningen, Groningen, Netherlands; 17 Department of Surgery, Meander Medical Centre, Amersfoort, Netherlands; 18 Department of Colorectal Surgery, Manchester University NHS Foundation Trust, Manchester, UK; 19 Department of Research and Development, Netherlands Comprehensive Cancer Organization, Eindhoven, Netherlands; 20 Department of Surgical Oncology, Centre Hospitalier Lyon Sud, Pierre-Bénite, France; 21 Department of Medical Oncology, University Medical Centre Utrecht, Utrecht, Netherlands; 22 Department of Medical Oncology, Erasmus MC Cancer Institute, Rotterdam, Netherlands; 23 Department of Surgery, Radboud University Medical Center, Nijmegen, Netherlands; 24 Centre for Colorectal Disease, St. Vincent’s University Hospital for Colorectal Disease, County Dublin, Ireland; 25 Department of Surgery, Amsterdam University Medical Centres, Amsterdam, Netherlands

**Keywords:** Colorectal surgery, Delphi Technique, ONCOLOGY, Treatment Outcome

## Abstract

**Abstract:**

**Introduction:**

Cancer recurrence is a pivotal outcome after treatment with curative intent for colon cancer patients, as it is related to worse survival, the subsequent need for further treatment and the associated use of healthcare resources. However, at present there is no consensus regarding the definition of locoregional recurrence of colon cancer. This contributes to variability in reporting outcomes and clinical management, limiting comparability across registries and clinical studies. This study aims to establish a consensus definition of locoregional recurrence using the Delphi method.

**Methods and analysis:**

We will conduct a worldwide Delphi consensus study among international experts involved in the treatment of colon cancer and recurrence (surgeons, medical oncologists, radiologists and pathologists). Conceptual elements of the survey were identified through literature exploration and concern: (i) anatomical locations, (ii) diagnostic criteria, (iii) resection margins and (iv) disease-free interval. Participants will be asked to rate their agreement on statements regarding the definition of locoregional recurrence of colon cancer. After the first round, we will analyse the data to determine which items have reached consensus for inclusion/exclusion and develop a second survey including items that did not reach consensus and any new items suggested by participants.

**Ethics and dissemination:**

The Dutch Medical Research Involving Human Subjects Act (WMO) does not apply to this study, as it does not involve patient participation. Participation is restricted to clinical health professionals. Results will be disseminated via communication to relevant societies in this field, presentations on (inter)national meetings and peer-reviewed publication.

**STRENGTHS AND LIMITATIONS OF THIS STUDY**

- We propose to address the current limitations in research on locoregional recurrence with the Delphi method that will allow for consistency in reporting and improve comparability.
- This study will involve experts from multiple countries worldwide, supporting the establishment of a definition with worldwide applicability.
- The composition of the expert panel may influence the outcomes, particularly if certain regions, practice settings or professional backgrounds are underrepresented.

## Introduction

 Colon cancer is the third most common cancer worldwide, and disease recurrence is the leading cause of mortality in patients with stage I–III colon cancer.^1 2^ Moreover, recurrence is related to the subsequent need for further treatment and the associated use of healthcare resources.^3^ Despite the fact that recurrence is a key outcome measure in clinical studies, a standardised definition and classification of locoregional recurrence of colon cancer is lacking in the literature and in international guidelines.^4^

Recently, three systematic reviews have been performed investigating the treatment and outcomes of locoregional recurrence in colon cancer, providing an overview of the different anatomical sites classified as locoregional recurrence.^5^,^7^ It is demonstrated that definitions of locoregional recurrence vary substantially between studies, with some not providing a definition at all or with limited anatomical specificity.^8^,^11^ While locoregional and distant recurrences are commonly reported as distinct entities, the boundary between these two remains unclear. For rectal cancer, the anatomical sites classified as locoregional recurrence have been defined as confined to the pelvic cavity, though not unambiguously.^12^,^14^ In contrast, definitions of locoregional recurrence of colon cancer range from strict inclusion of peri-anastomotic or tumour bed recurrences to broader definitions that also encompass lymph node recurrences in the adjacent mesocolon or abdominal wall as well as peritoneal metastases.^15^,^18^

The term ‘recurrence’ implies the reappearance of a tumour after a period during which no (macroscopically visible) disease was detectable. However, many studies do not consider the timing of recurrence in relation to the resection of the primary tumour.^2 16 19^ Although clinical guidelines report both disease progression and recurrence, an established disease-free interval to distinguish between the two is lacking.^4^ Another discussion point in this context is the completeness of the primary tumour resection. In cases of macroscopic residual disease (R2 resection), a disease-free interval is not possible by definition. Nevertheless, some studies include such cases in recurrence analyses.^18 20^ Furthermore, variability exists among studies regarding microscopic residual disease (R1 resection), which is a recognised risk factor for recurrence but does not inevitably result in recurrent disease, representing an additional challenge in defining recurrence.^16 19 21^

Additional heterogeneity exists in diagnostic approaches used to confirm recurrence diagnoses. Histopathological examination is the gold standard for diagnosing primary (colon) cancer but is not always feasible or appropriate when a recurrence is suspected.^22^ Insisting on histopathological confirmation of a recurrence has disadvantages related to invasiveness and the associated risk of complications of biopsies, sampling error with false-negative results that can delay proper management and technical difficulties in obtaining tissue from certain intra-abdominal areas.^23^ Consequently, a recurrence diagnosis is imaging-based in several studies, with or without pathology or elevated serum marker elevation.^19 24 25^ However, the diagnostic performance of cross-sectional imaging modalities such as CT and MRI is limited by difficulties in distinguishing benign postoperative or post-treatment changes from subtle signs of recurrence, as well as by known mismatch rates between imaging findings and pathological confirmation in complex cases.

The lack of a standardised definition limits transparency and comparability of study results. Given the substantial heterogeneity in definitions and the variety of clinical and methodological considerations involved, establishing a consensus statement based solely on the existing literature is not feasible. Therefore, developing a standardised definition requires structured expert judgement to interpret the available evidence, reconcile differing clinical perspectives and agree on acceptable conceptual boundaries. Accordingly, this Delphi study aims to establish a worldwide consensus-based definition of locoregional recurrence after treatment with curative intent for primary colon cancer. This would facilitate international dialogue, streamline research methodologies, improve the measurement of treatment effects and enhance comparability between studies focusing on colon cancer recurrence.

## Methods and design

### Study design

**Figure 1:**
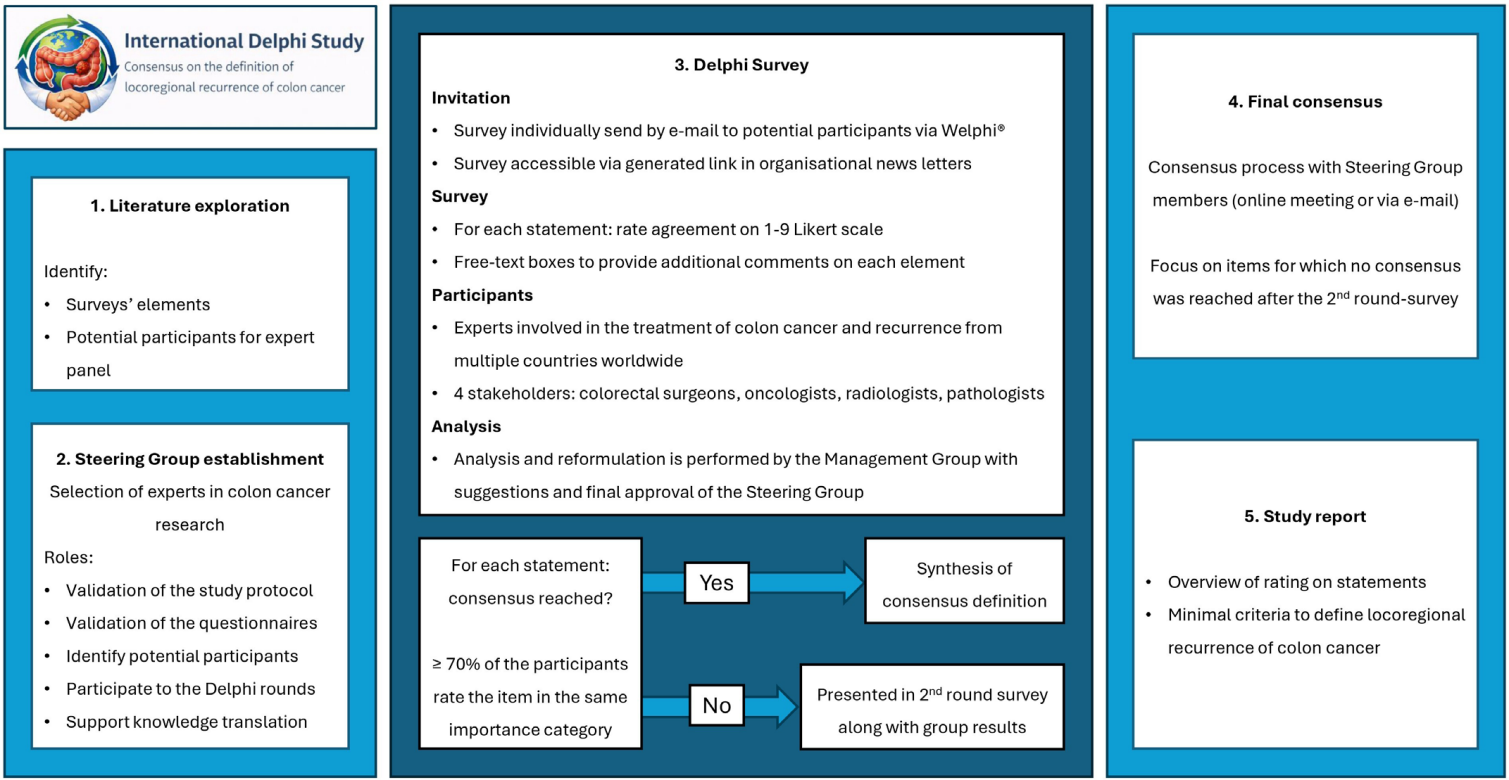
Overview of the study process.

We will conduct an intercontinental Delphi study among international experts in colon cancer. The Delphi method is based on a structured process for gathering and condensing knowledge fromstakeholder groups by means of a series of questionnaires.^26^ An overview of the study process is provided in figure 1. The first Delphi survey is scheduled to be launched in April 2026. Guidelines as proposed by the Grading of Recommendations Assessment, Development and Evaluation (GRADE) working group, Core Outcome Measures in Effectiveness Trials initiative and ACcurateCOnsensusReporting Document guidelines will be followed.^27^,^29^

### Research group

The study management group (PJT, JBT, JPMD, AEP and ER) coordinates the overall project and will hold regular meetings to monitor study progress. The management group has invited experts from multiple European countries to participate as members of the steering group, ensuring heterogeneous representation while taking feasibility related to time zones into account (AA-S, EA, SB, CAB, JGB, WC, ECJC, DH, IHJTdH, OG, BG, HP, JMLR, MR, HMWV, JHWdW, HLvW and DCW). The steering group has reviewed the study protocol and will be involved in the development of the first-round questionnaire. Throughout the process, the steering group will be consulted for advice on incorporating results and developing subsequent questionnaires.

### Literature exploration

To inform the Delphi study, a targeted literature exploration was conducted to gain insight into how locoregional recurrence of colon cancer is defined in the existing literature, based on 28 studies.^29 10 15^,^21 23^ This exploration consisted of mapping existing definitions and identifying their underlying conceptual elements. Further details on the literature exploration are provided in online supplemental material. Based on this literature exploration and in accordance with the steering group, the following key conceptual elements were identified: anatomical location, resection margin, diagnostic criteria and disease-free interval. These elements served as the basis for the statements that will be presented to participants in the first questionnaire.

### Participants

A variety of stakeholders will be invited to participate in this worldwide Delphi study to ensure the definition of locoregional recurrence reflects the opinion of the international community. Participants will be organised into stakeholder groups based on their areas of expertise, including colorectal surgeons, oncologists, radiologists and pathologists. In the first Delphi round, participants will be asked to report the number of years of experience within their respective specialty.

### Patient and public involvement

Patients were not involved in the design, conduct, reporting or dissemination plans of this research and will not be included as a stakeholder group. While recurrence is undoubtedly a pivotal outcome for patients treated for colon cancer, the definition of recurrence is a clinical determination intended for use in a clinical setting and research by clinical healthcare professionals, rather than by patients.

### Recruitment

There is no predefined threshold for the minimal panel size required for a Delphi study.^27^ We aim to recruit at least 75 colorectal surgeons, 25 oncologists, 25 radiologists and 10 pathologists. The Delphi survey will be conducted using Welphi, which allows participation via personalised email invitations as well as open access through a survey link. Once participants start completing the survey, they will be presented with an overview of the study objectives, detailed instructions, the literature exploration and information emphasising the importance of completing all Delphi rounds.

Potential participants will be identified using several recruitment strategies. First, healthcare professionals who have conducted clinical trials on colon cancer treatment within the past 5 years will be identified through searches of the ClinicalTrials.gov database using the term ‘colon cancer’, as well as research groups involved in studies identified during the literature review. Second, steering group members will be asked to provide contact details of experts within their professional networks. Third, steering group members will be encouraged to use their roles within relevant national and international colorectal cancer associations to disseminate the survey link via organisational newsletters or other professional communication channels. Finally, at the end of the questionnaire, participants will be asked to invite additional experts from their professional networks to participate via a dedicated link.

ER and AEP will be responsible for panellist selection. Eligibility criteria for expert panellists will be: being a consultant working as a colorectal surgeon, medical oncologist, radiologist or pathologist and having at least 3 years of clinical experience as a consultant, with no upper limit. Members of the steering group will also be eligible to participate in the Delphi process. Members of the management group will not be eligible to participate to prevent potential conflicts of interest and to safeguard methodological independence.

Participants will have 3 weeks to complete each round, with reminders sent to non-respondents after 7 and 14 days to maximise the response rate. Participation is voluntary, and participants may withdraw at any time without providing a reason. Survey responses are processed anonymously and will be analysed at a group level only. No financial compensation will be offered to participants.

### Delphi survey

The questionnaires will be developed by the management group, incorporating suggestions and requiring final approval from the steering group. The Delphi survey will be formulated in English and accessible simultaneously to respondents from all participating countries. Participants will be asked to rate their agreement on each statement on a nine-point Likert scale. A score of 7–9 indicates that a domain is considered critically important for the definition of locoregional recurrence, 4–6 indicates the outcome is considered important but not critical, and 1–3 indicates the component is of low importance for the definition of locoregional recurrence. In addition, it will be possible to select “unable to score/not my area of expertise” for each question, intended for respondents who do not feel equipped to score certain components.

### Definition of consensus

“Consensus-in” will be defined as:

- At least 70% of the participants (excluding those selecting “Not my area of expertise”) rated the component as 7–9 and less than 15% rated the variable as 1–3.
- At least 85% of participants (excluding those selecting “Not my area of expertise”) within one stakeholder group rate the component as 7–9 ‘consensus-in’. This entails that the components that are only of interest to one stakeholder group can also be included.

“Consensus-out” will be defined as:

- At least 70% of the participants (excluding those selecting “Not my area of expertise”) rated the component as 1–3 and less than 15% of participants rated 7–9.
- Components that do not meet any of these criteria will be classified as ‘no consensus’.

### First round

Participants will be asked to provide basic demographic characteristics (eg, age, sex and country of practice), workplace (academic hospital, teaching non-academic hospital and non-teaching hospital), specialty (eg, surgeon, oncologist, radiologist and pathologist) and years of experience as a certified specialist. Several statements regarding each conceptual component identified through the literature exploration will be presented to the participants for consideration. Participants will be asked to score the statements according to their perceived agreement on the definition of locoregional recurrence. In the first round, participants may propose additional components not included in the initial list. After analysis of the first round, the study management group and steering group will decide whether components suggested by respondents should be included in further rounds. In addition, questions will be rephrased if misinterpretation is suspected.

### Second round

All participants who complete the first round will be invited to participate in the second round. Statements classified as ‘consensus out’ will be excluded from the second round, whereas those classified as “consensus in” will be presented again. An overview of the included and excluded statements will be provided. In accordance with the Delphi principle, anonymised responses from round one will be shared. Participants will receive histograms displaying the distribution of responses for each stakeholder group, together with a reminder of their individual response from round one. In round two, participants will be asked to rate the statements using the same method as in round one. The results of the second round will be analysed per stakeholder group and for the entire cohort using descriptive statistics, applying the same criteria for ‘consensus in’ and ‘consensus out’ as in the first Delphi round. A stratified analysis may be performed to assess skewness from divergent opinions within a single country or specialty.

### Final consensus

Following completion of the Delphi survey rounds, a final consensus process will be conducted with members of the steering group. This may take the form of an online consensus meeting or, if sufficient participation cannot be achieved, an asynchronous procedure by email, allowing steering group members to provide written feedback. This process will focus on items for which consensus was not achieved during the Delphi rounds. For each such item, a summary of the Delphi results will be provided using descriptive statistics, including the median rating, range of scores and relevant comments from the expert panel. The steering group will then discuss each item to explore reasons for disagreement and clarify remaining ambiguities, followed by a final decision regarding its inclusion in the definition of locoregional recurrence. Possible outcomes include: (A) inclusion of the item; (B) exclusion of the item; (C) insufficient available evidence and expert opinion to support inclusion or exclusion of the item. A summary of this final consensus process, including decisions made and their underlying rationale, will be documented and shared with the full Delphi expert panel.

## Ethics and dissemination

The Dutch Medical Research Involving Human Subjects Act (WMO) does not apply to this study, as it does not involve patient participation. Participation is restricted to clinical healthcare professionals. Results will be disseminated via communication to relevant societies in this field, presentations on (inter)national meetings and peer-reviewed publication.

## Supplementary material

10.1136/bmjopen-2026-118765 online supplemental file 1
