# Supplementary material for: Consensus on the definition of locoregional recurrence of colon cancer: protocol for an international Delphi study
Source: BMJ Open. 2026 Jun 30;16(6):e118765. doi: 10.1136/bmjopen-2026-118765 (PMC13331237; doi:10.1136/bmjopen-2026-118765)
Supplement: online supplemental file 1 [file bmjopen-16-6-s001.docx]

[Supplementary material]

Literature exploration informing Delphi item development

# Aim

The aim of this literature exploration was to identify how locoregional recurrence after colon cancer surgery is defined in the existing literature. The findings were used exclusively to inform generation for the first Delphi questionnaire.

# Search strategy and data extraction

A targeted literature exploration was conducted based on three recent systematic reviews that provided an overview of the definitions used for locoregional recurrence of colon cancer. One of these reviews was conducted by two authors of the present research group and included studies published between 2010 and 2024.^7^ The two additional systematic reviews were identified during the literature search performed for that review.^5,6^ These reviews also included studies published prior to 2010, thereby allowing exploration of earlier definitions. All primary studies included in the three systematic reviews were screened for eligibility. Studies that did not provide an explicit definition of locoregional recurrence were excluded. Reference list screening of the included reviews and eligible primary studies was subsequently performed to identify any additional relevant studies.

During screening, both reviewers applied the same predefined eligibility criterion: studies were included only if they explicitly reported a definition of locoregional recurrence after colon cancer surgery; studies without such a definition were excluded. The definitions identified in the included studies were subsequently categorised according to their conceptual components: anatomical location, time interval, resection margin status of the primary tumour, and diagnostic modality.

Two reviewers independently extracted data using a predefined data extraction form. Extracted variables included publication year, whether the study population consisted exclusively of patients with colon cancer or also included rectal cancer patients, whether a definition of recurrence was provided, and which conceptual components were included in that definition, together with further specification of the conceptual component. A conceptual component was also considered present when it was incorporated as in or exclusion criterion of the study. Any disagreements were resolved through consensus discussion. The proportion of cases requiring consensus was not recorded.

# Overview of identified conceptual elements

In total, 28 studies published between 1976 and 2024 were included.^2,9,10,15-21,23-25,30-44^ Sixteen studies included patients with colon cancer exclusively, while eight studies included patients with colon and rectal cancer (Table S1).

Across the included studies, substantial heterogeneity was observed in the conceptual components used to define locoregional recurrence. Anatomical location was the most consistently specified component, although the extent and boundaries of what was considered locoregional varied considerably. In addition to anatomical location, some studies incorporated further elements in their definitions, including a disease-free interval, resection margin status, and the diagnostic modalities used to confirm recurrence.

## **Anatomical location of locoregional recurrence**

Locations classified as locoregional recurrence varied across included studies. Some studies restrict locoregional recurrence to the peri-anastomotic region and tumour bed.^33,39^ Others also include lymph node recurrences in the adjacent colonic mesentery or the abdominal wall or pelvis.^25,34^ Besides, peritoneal recurrences were inconsistently considered as locoregional and distant recurrence.^18,24,36,40^

Furthermore, the level of anatomical specificity varied considerably. While some studies provided detailed listings of anatomical sites—such as regional lymph nodes defined according to TNM classification, para-aortic lymph nodes, cicatrix, or pelvic bone involvement—others used more general descriptions, including recurrence occurring ‘near the primary tumour site’, ‘within the field of previous surgery’, or ‘involving adjacent organs or structures’.^2,19,38^

## **Diagnostic modalities**

Diagnostic modalities used to confirm locoregional recurrence were reported in nine studies.^19,21,23-25,30,32,38,40^ In several studies, recurrence was confirmed either histologically or radiologically, depending on feasibility.^19,24,38^ One study mentioned colonoscopy, in addition to imaging, as a diagnostic modality; however, it did not specify whether colonoscopic findings were histopathologically confirmed.^40^ In another study, histological verification was waived when recurrence was considered sufficiently likely based on imaging findings discussed in a multidisciplinary team setting.^21^ Additional approaches included confirmation of recurrence based on clinical evidence of tumour regrowth, disease progression observed on serial imaging, or rising carcinoembryonic antigen levels when supported by radiological findings.^25,32,38^ Two studies noted that, in a subset of patients with recurrence, the diagnosis was considered obvious despite the absence of histopathological verification.^23,30^ While considerable variability existed in the diagnostic modalities used to confirm locoregional recurrence, none of the included studies explicitly required histopathological confirmation as a prerequisite for establishing recurrence diagnosis.

## **Resection margins**

A limited number of studies explicitly addressed resection margin status in their definition of locoregional recurrence. In some studies, recurrences following R2 resections were excluded.^16,21^ However, variability was observed with respect to R0 and R1 resections. Some studies limited their study cohort to patients with R0 resections^10,17,19,23,39^, whereas others also included R1 resections.^16,21^ In some studies, resection margin status was not incorporated into the formal definition of locoregional recurrence but used for stratification or subgroup analyses.^20,42^ Another study included only patients described as having undergone a “complete resection”, without clarifying whether this referred to macroscopic or microscopic completeness.^30^

## **Disease-free interval**

A limited number of studies considered the timing of recurrence following primary surgery. Two studies applied a minimum disease-free interval of six months.^17,32^ Another study stratified recurrences according to a disease-free interval of less than or greater than 24 months, but classified both categories as recurrence without applying a minimum disease-free interval.^18^ Two studies specifically mentioned ‘metachronous’ recurrence, implying a distinction from synchronous disease, but did not specify a time-based cut-off.^21,41^ In some other studies, the disease-free interval was reported descriptively but not used as a defining criterion.^31^

## **Other elements**

In addition to the commonly reported components, some studies incorporated other criteria when defining locoregional recurrence. One study required a recurrence to share the same histological or morphological characteristics as the primary tumour, in order to distinguish such a recurrence from a second primary malignancy.^21^ Several studies specified in their inclusion criteria that only patients treated with curative intent were included.

# Translation to Delphi questionnaire

Based on the various identified definitions used for locoregional recurrence, the following key conceptual elements were selected for inclusion in the Delphi questionnaire: anatomical locations, resection margins, diagnostic modalities, and disease-free interval. These elements will be the domains within which statements will be formulated and presented to the expert panel. The specific content and boundaries of each element will be explored through expert agreement on the proposed statements.

| Study (year), population | Components included in definition | Specification of anatomical locations |
| --- | --- | --- |
| Akiyoshi (2011)^18^, C | Anatomical locations, resection margins | Anastomotic, peri-anastomotic, mesentery, nodal, peritoneal, retroperitoneal |
| Andreoni (2007)^19^, CR | (Anatomical) locations, diagnostic modalities, resection margins | Within the field of previous surgery |
| Akgun (2018)^16^, C | Anatomical locations, resection margins | Anastomotic, in the abdomen (abdominal wall, retroperitoneum, mesentery, lymph nodes), pelvis |
| Bertelsen (2023)^21^, C | Anatomical locations, diagnostic modalities, resection margins, (disease-free interval) | Anastomotic, regional lymph nodes, infra-renal paraaortic lymph nodes, abdominal wall, cicatrix, peritoneum, retroperitoneum, tumour bed, pelvic bone with relation to resected colon segments |
| Bouvier (2024)^2^, CR | Anatomical locations | Original tumour site, anastomosis, regional lymph nodes according to TNM |
| Bowne (2005)^20^, C | Anatomical locations, resection margins | Anastomotic, mesentery, nodal, peritoneum, retroperitoneum |
| Cass (1976)^30^, CR | Anatomical locations, diagnostic modalities | Contiguous to operative area, abdominal incision, peritoneal implants |
| Chesney (2021)^31^, C | Anatomical locations | Anastomotic, nodal, peritoneal, retroperitoneal, abdominal wall, pelvis |
| Elferink (2012)^32^, C | Anatomical locations, diagnostic modalities, disease-free interval | In or nearby the primary site |
| Hallet (2014)^33^, C | (Anatomical) locations | Involving adjacent organ or structures |
| Harji (2013)^34^, C | Anatomical locations | Anastomotic, nodal, retroperitoneal, abdominal wall, pelvis |
| Harris (2002)^35^, C | Anatomical locations | Anastomotic, abdominal peritoneum, pelvic peritoneum |
| Huang (2024)^15^, C | Anatomical locations, disease-free interval | Anastomotic, mesentery/nodal, tumour bed |
| Jarrar (2020)^36^, C | Anatomical locations | Anastomotic, peritoneal, pelvis |
| Kogler (2014)^9^, CR | (Anatomical) locations | At the initial tumour site or in the area of tumour-associated lymphatic drainage or perianastomotic recurrences |
| Landmann (2005)^37^, C | Anatomical locations | Perianastomotic (mural disease), mesenteric (regional nodal disease), retroperitoneal (drop metastases, distant nodal disease, or residual transmural disease), peritoneal |
| Liska (2017)^38^, C | (Anatomical) locations, diagnostic modalities | Near the primary site |
| Manfredi (2006)^39^, C | (Anatomical) locations, resection margins | Tumour bed or in the bowel anastomosis |
| Obrand (1997)^23^, CR | Anatomical locations, diagnostic modalities, resection margins | Local: endoluminal at the anastomotic site. Regional: in the pelvis or in the region of resection |
| Park (2015)^40^, C | Anatomical locations, diagnostic modalities | Perianastomotic, mesenteric, peritoneal, retroperitoneal |
| Qaderi (2021)^41^, CR | (Anatomical) locations | Original tumour site, regional lymph nodes according to TNM |
| Ramphal (2018)^24^, CR | (Anatomical) locations, diagnostic modalities | Intraabdominally or within the pelvic cavity, not peritoneal |
| Read (2002)^25^, C | Anatomical locations, diagnostic modalities | Anastomosis or adjacent mesentery, peritoneum, retroperitoneum |
| Sjovall (2007)^10^, C | Anatomical locations, resection margins | Abdominal recurrences in non-parenchymal organs |
| Swartjes (2023)^42^, C | (Anatomical) locations | Near the site of the primary tumour, in lymph nodes that would classify as regional lymph nodes, or both |
| Stipa (1991)^43^, CR | Anatomical locations | At the suture line, regional nodes, adjoining structures, pelvis and perineum |
| Taylor (2001)^17^, C | (Anatomical) locations, resection margins, disease-free interval | Local: involving adjacent structures.  Nodal: isolated intra-abdominal nodal. |
| Willett (1984)^44^, C | Anatomical locations | Local: recurrence occurring within the tumour bed in adjacent organs by direct extension.  Regional: in regional nodal groups. Regional nodal groups include lymph nodes up to the take-off of the colic vessels. |

**Supplementary table 1.** Overview of conceptual elements used in the literature to define locoregional recurrence in colon cancer, including specification of anatomical locations classified as locoregional recurrence. C, study population consisted exclusively of colon cancer patients. CR, study population consisted of both colon and rectal cancer patients.
