# Supplementary material for: Consensus on the definition of locoregional recurrence of colon cancer: protocol for an international Delphi study
Source: BMJ Open. 2026 Jun 30;16(6):e118765. doi: 10.1136/bmjopen-2026-118765 (PMC13331237; doi:10.1136/bmjopen-2026-118765)
Supplement: Supplementary file 2 [file bmjopen-2026-118765.reviewer_comments.pdf]

PEER REVIEW HISTORY

BMJ Open publishes all reviews undertaken for accepted manuscripts. Reviewers are asked to complete a checklist review form and are provided with free text boxes to elaborate on their assessment. These free text comments are reproduced below.

ARTICLE DETAILS

Title (Provisional)

Consensus on the definition of locoregional recurrence of colon cancer: protocol for an international Delphi Study

Authors

Rademaker, Eva; Petersen, Anne; Angenete, Eva; Arjona-Sánchez, Alvaro; Benz, Stefan; Bertelsen, Claus Anders; Bloemen, Johanne G; Ceelen, Wim; Consten, Esther C.J.; Harji, Deena; de Hingh, Ignace; Glehen, O.; Griffiths, Ben; Perregaard, Helene; Roodhart, Jeanine M. L.; Rutegård, Martin; Verheul, Henk M.W.; de Wilt, J.H.W.(Hans); van Westreenen, Henderik L; Winter, Des; Tuynman, Jurriaan B; Derikx, Joep P.M.; Tanis, Pieter J

VERSION 1 - REVIEW

**Reviewer**	**1**
**Name**	**Faggioni, Lorenzo**
**Affiliation**	**University of Pisa**
**Date**	**25-Mar-2026**
**COI**	**None**

I have the following minor comments:

1) Introduction (page 6 lines 5-7). The issue of recurrence diagnosis being often based on imaging is a very relevant one and should be discussed in more detail (at least one dedicated sentence) with respect to the strengths and weaknesses of currently available imaging techniques (US, MRI, etc.). A specific mention should be made to the limitations of existing imaging modalities in differentiating benign findings (including post-treatment sequelae) vs subtle disease recurrence (which may also overlap with fibrotic changes, etc.) and the mismatch rate between imaging and pathology especially in such difficult cases.

2) Methods and design (Research group, page 6 lines 47-49). Please report the minimum and maximum degree of experience (e.g. in terms of years post-board certification and/or of subspecialty professional expertise) for the entire panel.

The same applies for the two literature reviewers mentioned in the Supplementary Material (Search Strategy And Data Extraction, page 1). If consensus assessment was needed in case of disagreement, in which proportion of cases was it performed?

3) Methods and design (Literature exploitation, pages 6-7). Please provide a brief explanation of the literature search criteria as adopted by the two literature reviewers - perhaps such details should be given in the Supplementary Material file.

4) Methods and design (Participants, page 7 lines 24-25). Why were patients not included as a stakeholder group?

5) Methods and design (Final consensus, page 9). If any inferential statistical methods are expected to be used to quantitative analyze data from the two Delphi rounds, please briefly describe them in a dedicated Statistical Analysis subsection.

**Reviewer**	**2**
**Name**	**Apte, Sameer**
**Affiliation**	**Ottawa Hospital, Surgical Oncology**
**Date**	**14-May-2026**
**COI**	**None**

This is a relatively straightforward protocol with buy-in from many international stakeholders who are experts in this field (strength). The protocol is well thought out, well-written, and uses agreed upon consensus design materials (ACCORD) which has been thoughtfully applied. The literature is very thorough, and clearly the authors have reviewed this literature in detail. With any delphi study - I always ask whether protocol publication is necessary, however, if the editors deem it so, then this one is publishable.

The topic of the review is an area of active controversy in the field, especially for surgeons and radiation oncologists. Having an agreed upon set of criteria is needed, and no group thus far has been able to accomplish this. If this group is able to do so, it would add to the sparse body of guidelines and context for those of us who manage locally advanced colorectal cancers.

VERSION 1 - AUTHOR RESPONSE

Reviewer: 1
Dr. Lorenzo Faggioni, University of Pisa
Comments to the Author:
I have the following minor comments:

1) Introduction (page 6 lines 5-7). The issue of recurrence diagnosis being often based

on imaging is a very relevant one and should be discussed in more detail (at least one dedicated sentence) with respect to the strengths and weaknesses of currently available imaging techniques (US, MRI, etc.). A specific mention should be made to the limitations of existing imaging modalities in differentiating benign findings (including post-treatment sequelae) vs subtle disease recurrence (which may also overlap with fibrotic changes, etc.) and the mismatch rate between imaging and pathology especially in such difficult cases.

Author's response: We thank the reviewer for this valuable suggestion. We agree that the limitations of imaging-based recurrence diagnosis warrant further clarification. We have therefore expanded the Introduction to include a dedicated sentence discussing the strengths and weaknesses of commonly used imaging modalities with specific reference to their challenges in distinguishing benign post-treatment changes from subtle locoregional recurrence and the known mismatch between imaging and pathological confirmation. This addition strengthens the rationale for the need for a consensus definition.

2) Methods and design (Research group, page 6 lines 47-49). Please report the minimum and maximum degree of experience (e.g. in terms of years post-board certification and/or of subspecialty professional expertise) for the entire panel.
The same applies for the two literature reviewers mentioned in the Supplementary Material (Search Strategy And Data Extraction, page 1). If consensus assessment was needed in case of disagreement, in which proportion of cases was it performed?

Author's response: Thank you for this comment. We agree that reporting the range of clinical experience is important. We have clarified in the Methods section that the minimum required experience for participation is three years as a certified consultant, and that no upper limit is imposed.

The literature screening and data extraction were performed independently by two reviewers. Although disagreements were resolved through consensus discussion, the exact proportion of cases requiring consensus was not recorded. We have clarified this in the Supplementary Material.

3) Methods and design (Literature exploitation, pages 6-7). Please provide a brief explanation of the literature search criteria as adopted by the two literature reviewers - perhaps such details should be given in the Supplementary Material file.

Author's response: Thank you for this comment. We have clarified in the Supplementary Material that studies were included only if they explicitly reported a definition of locoregional recurrence after colon cancer surgery, and that studies without such a definition were excluded. We also specify that the subsequent categorisation of

definitions (anatomical location, time interval, resection margin status, and diagnostic modality) reflects the data extraction framework.

4) Methods and design (Participants, page 7 lines 24-25). Why were patients not included as a stakeholder group?

Author's response: Thank you for this comment. We fully acknowledge that locoregional recurrence is highly relevant to patients, as they are directly affected by the diagnosis. However, the purpose of this Delphi study is to establish a clinically and diagnostically robust definition of locoregional recurrence after colon cancer surgery. Determining whether a finding represents residual disease, a true locoregional recurrence, or metastatic spread requires specialised surgical, radiological, pathological, and oncological expertise. For this reason, the definition itself must be developed by clinical experts who are responsible for diagnosing and managing these scenarios.

5) Methods and design (Final consensus, page 9). If any inferential statistical methods are expected to be used to quantitative analyze data from the two Delphi rounds, please briefly describe them in a dedicated Statistical Analysis subsection.

Author's response: Thank you for this comment. No inferential statistical methods are planned for the analysis of the Delphi rounds, as the aim of the study is to describe and summarise expert ratings rather than to test statistical hypotheses. We have added a statement that only descriptive statistics (median, range, and percentage agreement) will be used to summarise responses from each Delphi round.

Reviewer: 2
Dr. Sameer Apte, Ottawa Hospital, University of Ottawa
Comments to the Author:
This is a relatively straightforward protocol with buy-in from many international stakeholders who are experts in this field (strength). The protocol is well thought out, well-written, and uses agreed upon consensus design materials (ACCORD) which has been thoughtfully applied. The literature is very thorough, and clearly the authors have reviewed this literature in detail. With any delphi study - I always ask whether protocol publication is necessary, however, if the editors deem it so, then this one is publishable.

The topic of the review is an area of active controversy in the field, especially for surgeons and radiation oncologists. Having an agreed upon set of criteria is needed, and no group thus far has been able to accomplish this. If this group is able to do so, it would add to the sparse body of guidelines and context for those of us who manage locally advanced colorectal cancers.

Author’s response: We thank the reviewer for the positive feedback regarding the relevance, methodological quality, and clarity of our protocol. We appreciate the reviewer’s recognition of the need for consensus in this area and their supportive comments. As no specific revisions were requested, no changes were made in response to this comment.
